# Coumarin-Palladium(II) Complex Acts as a Potent and Non-Toxic Anticancer Agent against Pancreatic Carcinoma Cells

**DOI:** 10.3390/molecules27072115

**Published:** 2022-03-25

**Authors:** Aleksandra Krstic, Aleksandar Pavic, Edina Avdovic, Zoran Markovic, Milena Stevanovic, Isidora Petrovic

**Affiliations:** 1 Institute of Molecular Genetics and Genetic Engineering, University of Belgrade, Vojvode Stepe 444a, 11042 Belgrade, Serbia; akrstic@imgge.bg.ac.rs (A.K.); pavicaleksandarr@gmail.com (A.P.); milenastevanovic@imgge.bg.ac.rs (M.S.); 2 Department of Science, Institute of Information Technologies, University of Kragujevac, Jovana Cvijica bb, 34000 Kragujevac, Serbia; edina.avdovic@pmf.kg.ac.rs (E.A.); zmarkovic@uni.kg.ac.rs (Z.M.); 3 Faculty of Biology, University of Belgrade, Studentski trg 16, 11000 Belgrade, Serbia; 4 Department of Chemical and Biological Sciences, Serbian Academy of Sciences and Arts, Kneza Mihaila 35, 11000 Belgrade, Serbia

**Keywords:** coumarin derivatives, coumarin palladium(II) complex, pancreatic carcinoma, anticancer

## Abstract

Pancreatic carcinoma still represents one of the most lethal malignant diseases in the world although some progress has been made in treating the disease in the past decades. Current multi-agent treatment options have improved the overall survival of patients, however, more effective treatment strategies are still needed. In this paper we have characterized the anticancer potential of coumarin-palladium(II) complex against pancreatic carcinoma cells. Cells viability, colony formation and migratory potential of pancreatic carcinoma cells were assessed in vitro, followed by evaluation of apoptosis induction and in vivo testing on zebrafish. Presented results showed remarkable reduction in pancreatic carcinoma cells growth both in vitro and in vivo, being effective at micromolar concentrations (0.5 μM). Treatments induced apoptosis, increased *BAX*/*BCL-2* ratio and suppressed the expression of *SOX9* and *SOX18*, genes shown to be significantly up-regulated in pancreatic ductal adenocarcinoma. Importantly, treatments of the zebrafish-pancreatic adenocarcinoma xenografts resulted in significant reduction in tumor mass, without provoking any adverse toxic effects including hepatotoxicity. Presented results indicate the great potential of the tested compound and the perspective of its further development towards pancreatic cancer therapy.

## 1. Introduction

Pancreatic carcinoma is among the most lethal malignant diseases in the world. The estimated number of new cases for 2020 was 495,773 with 466,003 deaths across the world according to Global Cancer Observatory (https://gco.iarc.fr/, accessed on 15 January 2022). Incidence and mortality of pancreatic carcinoma are nearly identical and are increasing in the past decades. Five year survival rate is about 6% and this varies a little between developed and developing countries [1,2].

Pancreatic ductal adenocarcinoma (PDAC) is the most prevalent type of pancreatic carcinoma and represents malignancy of exocrine pancreas, while endocrine malignancies are rare. This disease causes only a few non-specific symptoms contributing to late diagnosis, usually when the disease is in an advanced stage and with rare possibility for surgical resection [2]. Even for patients who are diagnosed with localized, resectable tumors, the prognosis is poor with less than 20% surviving five years following surgery [2]. Another problem is well developed pancreatic desmoplastic stroma, which contributes to the chemoresistance of a tumor by limiting drug delivery and creating a tumor promoting microenvironment [3,4]. The current multi-drug treatment options, like treatment with modified FOLFIRINOX (Oxaliplatin, Leucovorin, Irinotecan, Fluorouracil) have improved the long-term survival of patients with pancreatic carcinoma, however more effective systemic therapies and novel treatment strategies are urgently needed [5]. Therefore, better understanding of the molecular biology of pancreatic carcinoma and a development of novel therapeutic approaches can help in improving the quality of life and survival of patients.

**Figure 1:**
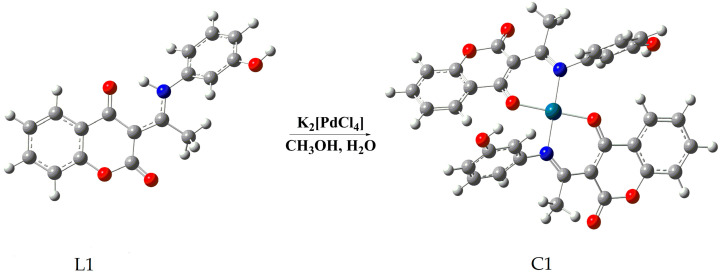
Schematic representation of the general procedure for the synthesis of complex Bis (3-(1-((3-hydroxyphenyl) amino)ethylidene) chroman-2,4-dione-palladium(II) complex (C1) obtained by reaction of 3-(1-(3-hydroxyphenyl)amino)ethylidene)chroman-2,4-dione (L1) and potassium tetrachloropalladate (II) (K_2_[PdCl_4_]).

Coumarins are bioactive natural compounds with a large number of physiological activities such as anti-coagulant, anti-tumor, anti-viral, anti-inflammatory, antioxidant, anti-microbial effects, as well as enzyme inhibition properties [6]. Several publications confirm an antitumor effect of various coumarin derivatives against PDAC and encourage further investigations on their antitumor potential [7,8,9,10]. Additionally, a large number of coumarin-based metal complexes have been synthesized in order to obtain molecules with more potent pharmacological activity, including those exhibiting anticancer properties [11]. Palladium(II) complexes present alternative candidates for antitumor metallobased drugs due to their structural and thermodynamic resemblances to the platinum(II) complexes, which have been widely used in the treatment of various malignancies. Accordingly, there is growing interest in the synthesis and examination of biological effects of palladium(II)-coumarin-based complexes. Recently, we have published results on studying the biological activities of several newly synthesized 4-hydroxycoumarin bidentate ligands and their corresponding palladium(II) complexes [12]. The results pointed to selective antiradical activity of palladium(II) complexes towards •OH and -•OOH radicals and anti-ABTS (2,2′-Azino-bis(3-ethylbenzothiazoline-6-sulfonic acid) cation radical) activity comparable to that of ascorbate. The results also indicated the effect on the enzymatic activity of the antioxidative defense system. Importantly, in vitro cytotoxicity assay performed on different carcinoma cell lines and one healthy fibroblast cell line showed the strongest effect against pancreatic carcinoma cell line MIA PaCa-2, indicating some tumor specific activity [12]. This paper is a continuation of our previous study and includes a more detailed analysis of the antitumor effect of coumarin-palladium(II) complex, Bis(3-(1-((3-hydroxyphenyl)amino)ethylidene)chroman2,4-dione-palladium(II)), designated as complex C1 (Figure 1) against pancreatic carcinoma cells in vitro and in vivo.

## 2. Results

### 2.1. Coumarin-Palladium(II) Complex Potently Reduces Viability and Proliferation of Pancreatic Carcinoma Cells In Vitro

**Figure 2:**
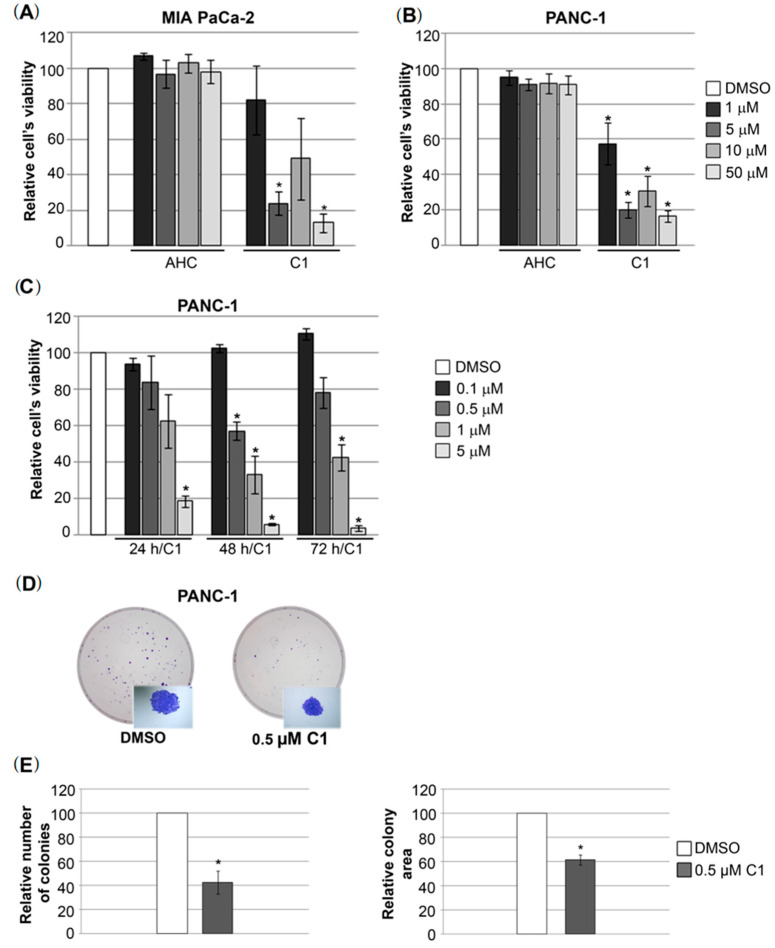
Effects of complex C1 on pancreatic carcinoma cells viability and potential to form colonies. (**A**) MIA-PaCa-2 cells were treated with DMSO as a vehicle, with various concentrations of initial compound, AHC and complex C1 for 24 h. The cell’s viability was calculated in relation to DMSO treated cell’s viability, which was set as 100%. Results were presented as the means ± SEM (standard error mean) of at least three independent experiments. Student’s *t*-test was used to calculate *p*-values (* *p* ≤ 0.05). (**B**) PANC-1 cells were treated with DMSO as a vehicle, various concentrations of initial compound, AHC and complex C1 for 24 h. The cell’s viability was calculated in relation to DMSO treated cell’s viability, which was set as 100%. Results were presented as the means ± SEM (standard error mean) of at least three independent experiments. Student’s *t*-test was used to calculate *p*-values (* *p* ≤ 0.05). (**C**) PANC-1 cells were treated with DMSO as a vehicle and with lower concentrations of complex C1 for 24 h, 48 h and 72 h. (**D**) Colony formation assay was performed on PANC-1 cells treated with DMSO as a vehicle or with 0.5 µM of complex C1 for a period of 10 days. Representative macroscopic images of colonies in Petri dish upon treatments are presented with corresponding microscopic images of colonies as an example at bottom right corners. Images were captured by digital camera. (**E**) Relative number of PANC-1 colonies and relative colony area were calculated in relation to these parameters measured in DMSO treated cells that were set as 100%. Results were presented as the means ± SEM of at least three independent experiments. Student’s *t*-test was used to calculate *p*-values (* *p* ≤ 0.05).

Previously, we have tested the cytotoxic activity of several newly synthesized coumarin ligands and their palladium(II) complexes and our results showed significant cytotoxic effect of specific complex C1 [12]. Its cytotoxicity was particularly prominent on the pancreatic carcinoma cell line MIA PaCa-2, which has been shown to be the most sensitive [12]. Bearing in mind the selective anticancer activity of the tested complexes towards pancreatic carcinoma cell line, we have further evaluated the cytotoxic effect of complex C1 by including another PDAC cell line (PANC-1) in this study. Comparative analysis of viability of MIA PaCa-2 (Figure 2A) and PANC-1 cells (Figure 2B) after treatment with increasing concentrations of complex C1 (1, 5, 10 and 50 μM) showed that both cell lines respond very similarly to treatment with selected concentrations over a period of 24 h. All tested C1 concentrations significantly reduced MIA PaCa-2 and PANC-1 cells viability compared to the treatment with the initial compound, 3-acetyl-4-hydroxycoumarin (AHC). The calculated IC_50_ value for complex C1 in PANC-1 cells was IC_50_ = 3.39 ± 0.31, which is similar to IC_50_ previously published for MIA-PaCa-2 cells (IC_50_ = 3 ± 0.11) [12]. However, PANC-1 cells showed a higher degree of sensitivity at lower concentrations. In particular, 1 μM led to reduction in MIA PaCa-2 cells viability to 82%, compared to PANC-1 where the same treatment led to significant reduction up to 57% (Figure 2A,B). Therefore, our goal was to test the effect of further reduction in concentration of complex C1 and to determine an effective dose that produces a biological response on PANC-1 cell.

We have treated PANC-1 cells with complex C1 concentrations below IC_50_ (1, 0.5 and 0.1 μM) and showed that with prolonged treatment time (48 h) both 1 μM and 0.5 μM significantly reduces PANC-1 cells viability to approximately 35% and 55%, respectively, while longer treatment (up to 72 h) had no additional effect (Figure 2C). Further reduction in the concentration (up to 0.1 μM) no longer led to a reduction in the cell’s viability (Figure 2C).

The clonogenic assay revealed that treatment with both 0.5 μM and 1 μM significantly impaired the capacity of PANC-1 cells to form colonies during the period of 10 days. In particular, treatment with 1 μM of complex C1 led to excessive reduction in cells proliferation and no colonies were formed during the prolonged treatment period of 10 days (data not shown). In parallel, lower concentration of 0.5 μM led to measurable reduction in the capacities of PANC-1 cells to form colonies from a single cell, during the same period (Figure 2D). We have observed both reduction in number of colonies (up to approximately 60%), and in colony size (approximately for 40%) (Figure 2E).

### 2.2. Coumarin-Palladium(II) Complex Impairs Migratory Potential of Pancreatic Carcinoma Cells In Vitro

**Figure 3:**
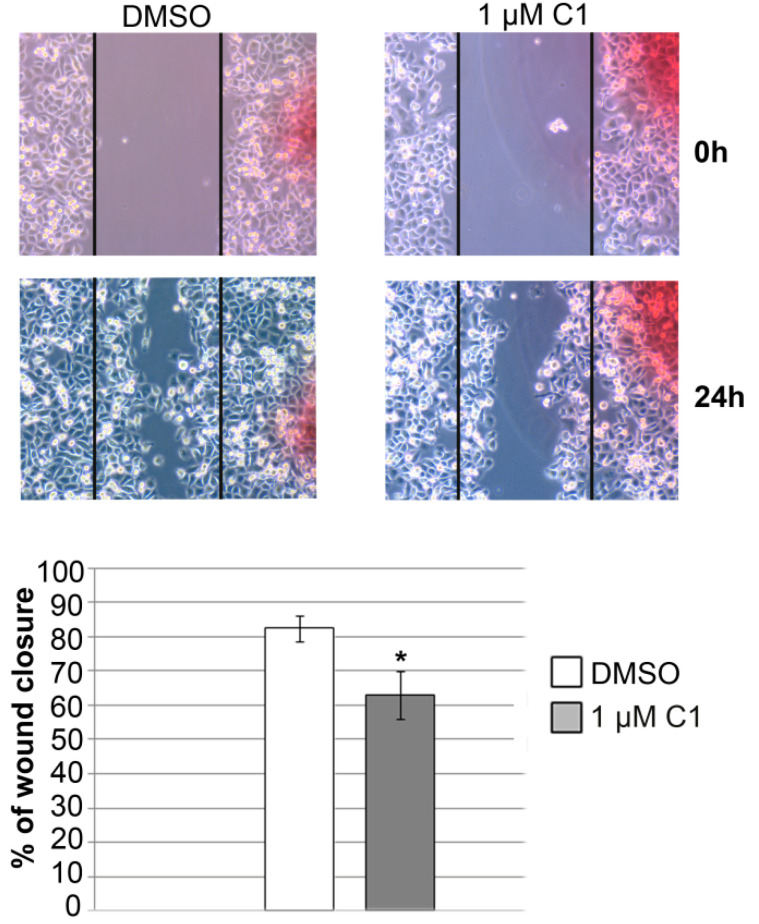
Effect of complex C1 on PANC-1 cell’s migratory potential. Cells were treated with DMSO or 1 µM complex C1, and scratch was made on 100% confluent cells in Petri dish. Gap was captured and measured after 0 h and 24 h (representative images are presented in upper panel). Cell migration was determined in 24 h time point by calculating percentage of wound closure compared to the gap width at 0 h that was set as 100%. Results were presented as the means ± SEM of at least three independent experiments. Student’s *t*-test was used to calculate *p*-values (* *p* ≤ 0.05).

The ability of cancer cells to migrate is closely related to their capacity to colonize distant organs [13]. Therefore we tested the migratory potential of PANC-1 cells upon treatment with complex C1, during 24 h. Results showed that treatment with complex C1 reduced the ability of PANC-1 cells to close the scratched area. Significant reduction in migratory potential is presented at Figure 3 revealing 80% wound closure in DMSO treated, compared to 60% wound closure in complex C1 treated cells upon 24 h, compared to the gap width at 0 h.

### 2.3. Apoptosis Is Induced upon the Treatment of PANC-1 with Coumarin-Palladium(II) Complex 

**Figure 4:**
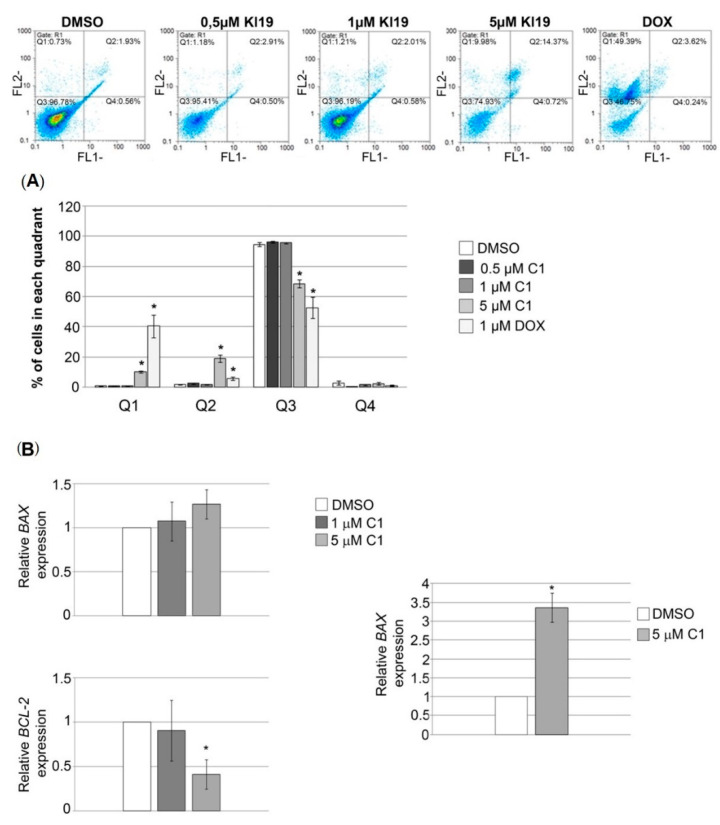
Pro-apoptotic effect of complex C1 on PANC-1 cells. (**A**) PANC-1 cells were treated with DMSO as a negative control or various concentrations of complex C1, upon treatment cells were collected and Annexin V-FITC assay was performed. The percentage of cells in each quadrant is presented as the means ± SEM of at least two (0.5 μM C1) or four (1 μM C1, 5 μM C1 and 1 μM dox) independent experiments. Mean values were compared with Student’s *t*-test, and *p* values that were evaluated compared to DMSO treatment are presented as * *p* ≤ 0.05. Each color in the legend corresponds to a bar presented on the histogram. Q1: necrotic cells; Q2: late apoptosis; Q3: living cells; Q4: early apoptosis. (**B**) Expression of *BAX* and *BCL-2* in PANC-1 cells upon treatment with complex C1 was determined by qRT-PCR. Relative expression of *BAX* and *BCL-2* was calculated as a percentage of expression in treated cells compared to their expression in cells treated with DMSO that was set as one. Results were presented as the means ± SEM of at least three independent experiments. Relative *BAX/BCL-2* ratio was calculated in each independent experiment and presented as means ± SEM of at least three experiments. Mean values were compared with Student’s *t*-test, and *p* values that were evaluated compared to DMSO treatment are presented as * *p* ≤ 0.05.

We aimed to investigate whether reduction in PANC-1 cells viability and proliferation is related to an increase in apoptotic events in these cells. Annexin V-FITC assay was used to assess apoptotic and necrotic cell death incidence upon treatment of PANC-1 cells with various concentrations of complex C1 (0.5, 1 and 5 μM). As presented at Figure 4A, we observed a gradual increase in the number of cells in apoptosis with the most prominent effect after application of the highest concentration used. After treatment with 5 µM complex C1, total percent of cells in necrotic and late apoptotic phase together was ten times higher compared to DMSO treatment. In particular, approximately 10% of cells were in necrotic and 20% of cells in late apoptotic phase upon treatment with 5 μM complex C1, compared to 0.7% and 2%, respectively, in DMSO treated cells. When compared to the well-known chemotherapeutic drug doxorubicin, complex C1 induced four times lower incidence of cell death by necrosis.

Since p53 tumor suppressor is mutated in majority of PDACs (50–75%), including PANC-1 cell line [14], we further investigated whether the changes in the BAX/BCL-2 ratio was involved in the p53 independent complex C1-induced apoptosis of PANC-1 cells. As presented at Figure 4B, complex C1 induced changes in BAX/BCL-2 ratio in PANC-1 cells showing approximately a 3.2-fold increase compared to the cells treated with vehicle. We observed apoptosis that is, at least in part, governed by an increase in BAX and decrease in BCL-2 expression in PANC-1 p53 mutated background.

### 2.4. Coumarin-Palladium(II) Complex Significantly Reduces SOX18 and SOX9 Expression in Pancreatic Carcinoma Cells In Vitro

**Figure 5:**
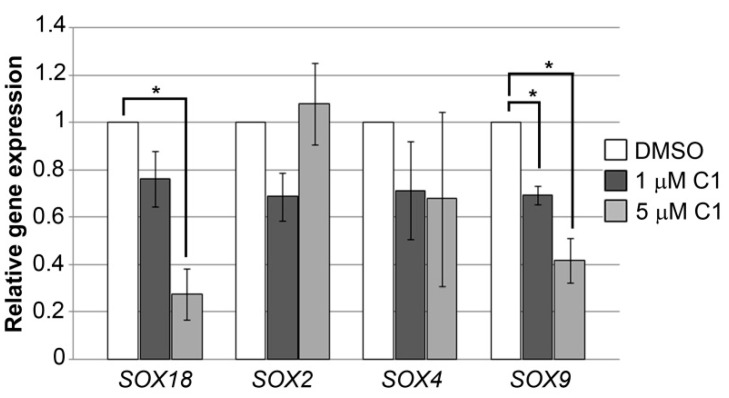
Effect of complex C1 on selected SOX genes expression. PANC-1 cells were treated with DMSO as a negative control and two concentrations of complex C1 (1 μM and 5 μM) and 24 h upon treatment cells were collected for RNA isolation followed by qRT-PCR. Relative expression of each *SOX* gene was calculated as a percentage of their expression in treated cells compared to their expression in cells treated with DMSO that was set as one. Results were presented as the means ± SEM of at least three independent experiments. Student’s *t*-test was used to calculate *p*-values (* *p* ≤ 0.05).

Each stage in the progression of pancreatic carcinoma is well characterized by multiple alterations in gene expression and signaling pathways, including the expression of the members of SOX family of transcription factors. So far, several *SOX* genes (*SOX4*, *SOX9*, *SOX10*, *SOX11*, and *SOX12*) have been shown to be enriched in the developing human pancreas, while some also remain actively expressed in adult pancreatic islets (*SOX4* and *SOX9*) and are reactivated during the initiation of pancreatic ductal adenocarcinoma (*SOX4*, *SOX9* and *SOX18*) or involved in therapy resistance (*SOX2*) [15,16,17,18,19]. In this paper we presented results regarding the effect of complex C1 on *SOX2*, *SOX4*, *SOX9* and *SOX18* genes expression upon treatment of PANC-1 cells. Two different concentrations of complex C1, 1 μM and 5 μM, were used and relative expressions of selected genes 24 h upon treatment are presented at Figure 5.

Complex C1 led to prominent reduction in *SOX9* and *SOX18* genes expression in PANC-1 cells. In particular, significant reduction was observed when 5 μM concentration was used leading to a decrease in *SOX9* expression up to 2.4-fold and *SOX18* expression up to 3.7-fold. A significant reduction in *SOX9* gene expression was observed even at 1 μM concentration. For the other genes and concentrations analyzed, we did not detect consistent effects on the trend in the expression pattern upon treatments with C1, and consequently there was no statistical significance. As for the expression of the *SOX2* gene in particular, inconsistency in terms of dose dependence was also detected, although overall these changes were not statistically significant.

This is the first time results of the inhibitory effect of any coumarin derivative on the expression of SOX genes that have previously shown to exhibit pro-oncogenic activities in pancreatic carcinoma cells have been presented. This result additionally confirms the importance of further investigation of complex C1 potential as an anticancer drug.

### 2.5. Anticancer Activity of Coumarin-Palladium(II) Complex in the Zebrafish Xenograft Model of Pancreatic Carcinoma

**Figure 6:**
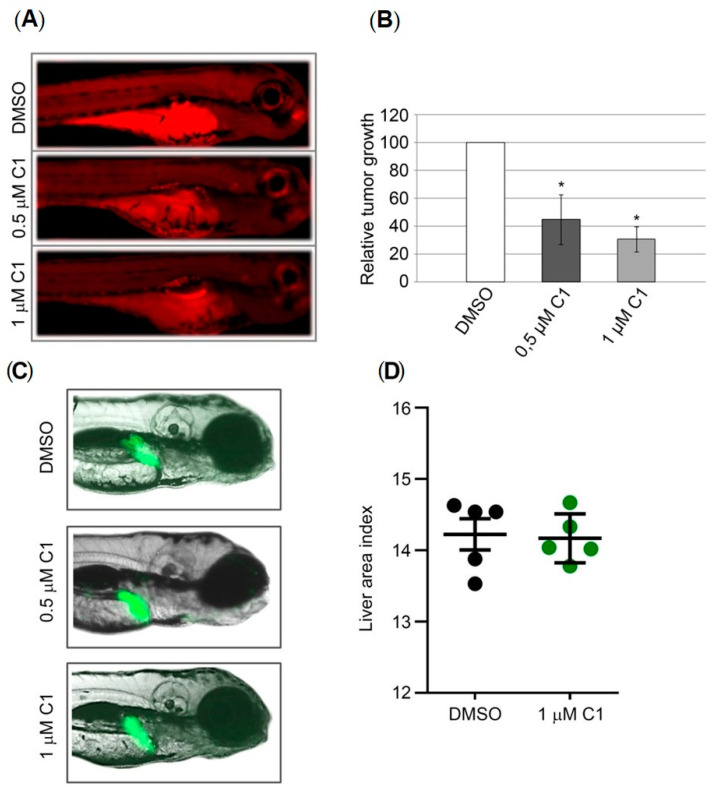
Anticancer activity of complex C1 against human PANC-1 cells in zebrafish xenografts and in vivo hepatotoxicity test. *Wt* xenografts (*n* = 30 per treatment in each independent experiment) were exposed to complex C1 at doses 0.5 and 1 μM, and analyzed three days post injection. (**A**) Representative fluorescent microscopy images. The applied treatments markedly reduced the tumor growth. (**B**) Quantification of tumor growth reduction. The relative tumor growth in treated PANC-1 xenografts was calculated as a percentage of tumor growth in xenografts treated with DMSO that was set as 100%. Mean values are presented ± SEM and Student’s *t*-test was used to calculate *p*-values (* *p* ≤ 0.05). (**C**) Hepatotoxicity was assessed according to the liver fluorescence and size (**D**) Hepatotoxicity presented as a dot chart of the liver area index. Experiment was performed using 10 embryos per concentration. The liver area index was determined for five randomly selected embryos by ImageJ program.

Our next step in the investigation of anticancer activity of complex C1 was an in vivo study using the zebrafish-PANC-1 xenograft model. Zebrafish xenografts serve as a platform for translational research of human carcinomas demonstrating crucial hallmarks of cancer biology, such as tumor cells proliferation, dissemination, metastasis and tumor angiogenesis [20]. Accordingly, PANC-1 cells were fluorescently labelled and injected into the yolk sack of *wt* zebrafish embryos, and at three days post injection (dpi), xenografts were processed for fluorescence microscopy evaluating the effects of the applied compound on the growth of the tumor mass and cancer cells dissemination. Our results show that treatment with complex C1 significantly inhibited pancreatic carcinoma cell growth in zebrafish xenografts (Figure 6A). This effect was observed at concentrations of 1 and 0.5 μM that represents 1/3 × IC_50_ and 1/6 × IC_50_ values, respectively, determined previously in vitro. Importantly, both concentrations had no adverse developmental effect and all xenografts survived without side effects. This is in accordance with our previously published data showing no embriotoxicity of complex C1 at concentration below 5 μM [12]. Relative reduction in tumor growth quantified by changes in fluorescence signal is presented at Figure 6B showing significant reduction in tumor mass by approximately 60% and 70% upon treatment with 0.5 μM and 1 μM, respectively. 

### 2.6. Hepatotoxicity Assessment

Keeping in mind that hepatotoxicity represents one of the most common adverse effects of anticancer drugs of clinical relevance, complex C1 was examined for a possible hepatotoxicity. In this assay, transgenic *Tg*(-2,8*fabp10a*:EGFP) embryos with GFP-expressing liver cells were exposed to 1/3 × IC_50_ and 1/6 × IC_50_ values of tested compound, allowing us to visually explore its effect on the liver development and functioning in real time. Embryos have been treated in a period from 72 hpf, a stage when the liver is fully functional, vascularized and started metabolic transformation of absorbed compounds, up to 120 hpf. Data obtained in the hepatotoxicity assay revealed that the tested compound was not hepatotoxic at the effective doses (Figure 6C), since there is no significant difference in the liver area index (the ration between the liver area and embryonic lateral area × 100%) between the treated and the control groups (*p* = 0.834, *t*-test) (Figure 6D).

## 3. Discussion

Numerous studies have been carried out in the last decades attempting to identify an effective drug or combinatorial therapies for PDAC. Although Gemcitabine has been used for many years as one of the main chemotherapy drugs used to treat pancreatic carcinoma in all stages of the disease, less than 25% of the pancreatic carcinoma patients benefit from Gemcitabine treatment with median overall survival of only six months, mainly due to developed resistance within weeks of chemotherapy initiation [21]. The current multi-agent treatment options like modified FOLFIRINOX have improved the long-term survival of patients with pancreatic carcinoma, with median overall survival extended to 54.4 months [22,23]. Since such treatment represents highly toxic and life-threatening regimen, more successful systemic therapies and treatment strategies are still needed. 

One of the important criteria for a therapeutic drug is to exert minimum or no side-effects on normal body cells of patients undergoing chemotherapy. One obvious way to achieve this is by employing lower doses of drug, while maintaining its effectiveness. Our goal in testing the potential anticancer activity of complex C1 was to try to meet these criteria. Here we have presented results showing that complex C1, even at a concentration six times lower than IC_50_ (0.5 μM), still significantly reduces PANC-1 cells viability and proliferative capacity in prolonged treatment regimen. Lewis et al. published data regarding the antitumor effect of dicumarol at concentrations of 50 μM and above, both in standard and reduced serum culturing conditions [24]. Another group reported significant variations in cytotoxic activity of isoprenylated coumarin derivatives on pancreatic carcinoma cells in vitro, showing IC_50_ for PANC-1 at 9 μM in nutrient-deprived conditions and above 100 μM under nutrient-rich conditions [10]. On the other hand, Suresh Awale and co-workers showed highly potent selective cytotoxicity of 7-hydroxycoumarin derivative against PANC-1 cells under nutrient-deprived conditions, while in a colony forming assay under nutrient-rich culturing conditions, moderate reduction in colony forming was observed only at 50 μM [25]. In comparison to previously published data regarding the effect of various coumarin-based compounds on pancreatic carcinoma cells, we have reached a significant scaling down of the inhibitory dose, showing IC_50_ at 3.3 μM and achieving significant reduction in cells viability and clonogenic potential at concentration as low as 0.5 μM. It should be noted here that complex C1 is coumarin palladium(II) complex and it has been reported that palladium(II) complexes have anti-tumor activity as much or even better than the cisplatin drugs [26,27]. Ulukaya and co-workers presented results of very potent anticancer activity of tested palladium(II) complex against breast carcinoma cells with IC_50_ values of 0.09 μM for MDA-MB-231 and 3.05 μM for MCF-7 cells [26]. The literature data reveals many coumarin-metal based complexes with antitumor activity, [28,29,30], however, this is the first report on coumarin palladium(II) complex anticancer activity against pancreatic adenocarcinoma cells.

In addition to the fact that these lower concentrations of complex C1 have shown to be effective in inhibiting the growth of tumor cells in vitro, our results also have revealed that these concentrations are very effective in reducing tumor growth in zebrafish xenograft model and, importantly, showing no signs of in vivo toxicity as was previously documented in toxicity testing [12]. We have shown that at a concentration of 5 μM of complex C1, the majority of the embryos exhibited normal development with few showing growth delay or, rarely, skeletal abnormalities, while at the concentration of 1 μM, no adverse developmental effects were detected [12]. Herein, we presented a detailed analysis of potential hepatotoxicity, testing sub-IC_50_ concentrations that have been shown to be highly effective against tumor growth in vivo. Hepatotoxicity is one of the commonly encountered drawbacks of clinically approved anticancer drugs and limits their long-term application in chemotherapy. Accordingly, it is important to point out that coumarin-palladium(II) complex C1 did not induce the liver failure in zebrafish model. This is in line with previously published results showing that palladium complexes were well-tolerated by the animals and less toxic as compared to cisplatin [26].

The cell death mechanisms have become an extremely important research subject when investigating the effect of potential therapeutics in the treatment of cancer. Broadly speaking, cancer cells regress or disappear following apoptosis induced by treatment, while necrosis causes the swelling of cells, inflammation of neighboring cells, hemorrhage and edema from the tumor tissue and is often recognized as a side effect of cancer treatments [31]. In addition, necrosis in the tumor microenvironment generally contributes to resistance to treatment with anticancer drugs and radiation [32]. Sato et al. identified cell death regulators that may play key roles in choosing between cell death by necrosis or apoptosis, stressing the importance of switching anticancer drug strategy from causing necrosis to inducing apoptosis [33]. In line with that, failing in inducing cancer cell apoptosis has often led to the clinical trial failure of several potential anticancer drugs. Johnstone et al. highlighted the importance of drug-induced apoptosis in tumor therapy and generally the role of apoptosis in treatment sensitivity [34]. Studies conducted on the anticancer activity of coumarin and its derivatives revealed that the mechanism of action of these compounds is generally caspase dependent apoptosis [35]. We have presented results showing that complex C1 is able to induce cell death in PANC-1 cells partially by inducing apoptosis, whereas doxorubicin in the same experimental conditions exerts pro-necrotic activity. BAX/BCL-2 ratio is among the most widely studied and reported protein expression pattern for assessment of apoptotic cancer cell death aimed to determine the eventual outcome, leading to apoptosis or survival [36]. As presented in this paper, treatment with complex C1 led to an increase in *BAX*/*BCL-2* ratio in PANC-1 cells. It is important to point out that the family of BCL-2-related pro-survival proteins besides BCL-2 includes BCL-X_L_, BCL-_W_, and MCL-1. These proteins inhibit cell death by sequestering the pro-apoptotic proteins BAX and BAK and by preventing their oligomerization [37]. While BAX and BAK are functionally redundant, meaning cells require one or the other for regulation of apoptosis, individual pro-survival proteins vary in abundance in different cell types, and also could be related to chemoresistance as is the case with BCL-X_L_ [38,39,40]. Therefore, additional investigation is needed in order to further elucidate an apoptotic pathway in PANC-1 upon C1 treatment. It is widely accepted that, in a variety of cell lines with wild-type p53, cell death triggered by apoptotic stimuli is accompanied by an increase in the BAX/BCL-2 ratio [41,42]. On the other hand, PANC-1 cells have mutated p53, and typically loss of p53 function correlates with multidrug resistance in many tumor types. However, it has been shown that cisplatin causes apoptosis in PANC-1 cells via a significant activation of BAX protein in the presence of p53 mutation [43]. In addition, it has been reported that radiation also induces apoptotic cell death in p53-mutated cell lines, accompanied by significant activation of BAX protein [44]. Therefore, our results confirm previous findings that PANC-1 cells could be triggered toward apoptosis regardless of inactive p53. Accordingly, complex C1 does not require p53 for its apoptotic function in PANC-1 cell.

Most PDAC is caused by sporadic somatic mutation and the sequencing of tumor samples has demonstrated that the majority of these tumors (over 90%) contain activating mutations in the KRAS proto-oncogene [45]. Constitutive KRAS activity leads to aberrant cell proliferation and differentiation as well as the activation of *SOX9* [46,47]. *SOX9* belongs to SRY-related box (*SOX*) gene family encoding a transcription factor that plays multiple and critical roles in PDAC initiation and progression [15,48]. It has been shown that *SOX9* is significantly overexpressed in high-grade pancreatic tumors and in chemotherapy-treated patients, compared to chemo-naive patients, and reduction in its expression is significant for the decrease in tumor growth and successes of combined chemotherapeutic regiments [49]. *SOX18* is another member of *SOX* family associated with pancreatic carcinoma, showing a high level of expression in PDAC tissue samples and correlation with poor prognosis [19]. Authors have shown that after knockdown of *SOX18* gene in PANC-1 and the SW1990 cell lines, the abilities of proliferation, migration and invasion were inhibited and the tumor growth was suppressed in vivo. Therefore the *SOX18* gene was marked as a promising target for PDAC therapy. In this paper we have presented the effect of complex C1 on selected *SOX* genes expression in PANC-1 cells, and shown that complex C1 successfully reduces both *SOX9* and *SOX18* expression. This particular effect of complex C1 is very important, since it is very difficult to manipulate the function of transcription factors, due to their localization in the nucleus. We hypothesize that the inhibition of *SOX9* and *SOX18* expression by complex C1 can be used to further sensitize the tumor for the conventional therapy. In particular, initial sensitivity of PDAC tumors to gemcitabine represents a window of opportunity and it could be combined with some novel agents that are also able to induce the apoptosis and preferably change the landscape of PDAC gene expression. Therefore, in our future efforts we will investigate the potential of the combined treatment of PDAC cells with complex C1 and some well-known chemotherapeutics.

In conclusion, the present study demonstrated that the complex C1 inhibited growth of PANC-1 cells, both in vitro and in vivo, and this is partially mediated by the induction of apoptosis. Additionally, the expression of *SOX9* and *SOX18* genes were down-regulated, showing the potential of complex C1 to target expression of these genes previously shown to be significantly up-regulated in PDAC samples. At the same time, complex C1 exhibited no hepatotoxicity in zebrafish, marking this compound as non-toxic and a safe agent, with promising anticancer activity. Future efforts will be directed towards verifying anticancer efficacy of complex C1 and its therapeutic potential in combination with known chemotherapeutics and elucidation of its molecular targets.

## 4. Materials and Methods

### 4.1. Chemistry

In this paper we have used: 3-acetyl-4-hydroxycoumarin (AHC), synthesized as previously described [50] in the reaction of 4-hydroxycoumarin (HC) and acetic acid with phosphoryl chloride as a catalyst. As detailed in a recent study [12] the C1 complex was made by a direct reaction between an equimolar quantity of potassium tetrachloropaladate (II) and a bidentate ligand of 3-(1-(3-Hydroxyphenyl)amino)ethylidene) chroman-2,4-dione. Elemental analysis, IR and NMR spectroscopy, and DFT analysis were used to identify the structure of the synthesized compound C1, as described earlier [12].

### 4.2. Cell Culture

Pancreatic carcinoma cell lines PANC-1 (ATCC, CRL-1469; Manassas, VA, USA) and MIA PaCa-2 (ATCC, CRL-1420, Manassas, VA, USA), were cultured in Dulbecco’s Modified Eagle’s medium (DMEM, Gibco, Waltham, MA, USA) supplemented with 10% fetal bovine serum (FBS, Gibco, Waltham, MA, USA) at 37 °C in 5% CO_2_ and passaged twice a week by trypsinization (0.5% trypsin-EDTA, Gibco, Waltham, MA, USA).

### 4.3. Cell Viability Assay

Cells (1 × 10^3^ per well) were seeded in 96-well plate and treated with various concentrations of complex C1 (0.1, 0.5, 1, 5, 10 and 50 µM) and DMSO for 24 h, 48 h and 72 h. Following treatments, cell viability was assessed using 3-(4,5-dimethylthiazol-2yl)-2,5-diphenyltetrazolium bromide (MTT) assay (Merck KGaA, Gernsheim, Germany). Final concentration of MTT solution added to cell cultures was 0.5 mg/mL and cells were incubated with solution for 1 h at 37 °C. After 1 h of incubation, MTT solution was removed and cells were lysed with DMSO (Serva Electrphoresis GmbH, Heidelberg, Germany). Reduction in a yellow tetrazolium salt to purple formazan crystals by metabolically viable cells was monitored using a microplate reader (Plate Reader Infinite 200 pro, Tecan, Mannedorf, Switzerland) at a wavelength of 620 nm. Cell’s viability was calculated in relation to DMSO treated cell’s viability.

### 4.4. Colony Formation Assay

PANC-1 cells (300 cells per well) were seeded in a twelve well plate and treated with complex C1 (0.5 µM and 1 µM concentration) for ten days. Treatment was renewed every two to three days. Following that, treatment colonies were stained with Cresyl violet. Colonies were captured using DM IL LED Inverted Microscope (Leica Microsystems, Wetzlar, Germany) and counted. The area of colonies was measured using image analyzing software Fiji (ImageJ 2.0.0.). The relative colony area and relative colony number were calculated in relation to these parameters, measured on DMSO treated cells.

### 4.5. Wound-Healing Assay

Cells (6 × 10^5^) were seeded in 35 mm dish and grown to confluence. Cell monolayer was scratched using 200 µL tip and treated with medium containing DMSO or 1 µM complex C1. Wounded area was captured at 0 h and 24 h post treatment using DM IL LED Inverted Microscope (Leica Microsystems, Wetzlar, Germany) and size of the gap and its rate of closure over time was quantified using Leica Application Suite V4.11.0. (Leica Microsystems, Wetzlar, Germany). Cell migration was determined at a 24 h time point by calculating the wound closure % where the gap width at zero hours was set to 100%.

### 4.6. Apoptosis Assay

Cells (5 × 10^5^) were seeded in 35 mm dish and treated after 24 h with DMSO, 0.5, 1 and 5 µM complex C1 and with 1 µM doxorubicin (Adriablastina, Pfizer, New York, NY, USA) as positive control. Then, 24 h after treatment, floating apoptotic cells and adherent cells were collected from the media and dish surface and resuspended in 1 × Annexin binding buffer at a final number of 1 × 10^6^ cells/mL. Cells were stained with 5 μL of Annexin V (Annexin V, Alexa Fluor1 488 conjugate, Invitrogen, Waltham, MA, USA) and 5 μL of propidium iodide (PI, Invitrogen, Waltham, MA, USA), mixed gently and incubated for 15 min in the dark at room temperature. Analyses were carried out by flow cytometer (CyFlow space, Partec, Munster, Germany) using FloMax 3.0 Ink software (CyFlow space, Partec, Munster, Germany) for cytometry. 

### 4.7. qRT-PCR

PANC-1 cells (5 × 10^5^) were seeded in a 35 mm dish and treated with various concentrations of complex C1 (1 and 5 µM) or DMSO as negative control for 24 h. Total cell RNA was isolated and treated with DNase I with DNA-Free kit (Invitrogen, Waltham, MA, USA). Isolated RNA was used for synthesis of cDNA by High Capacity cDNA Reverse Transcription Kit (Applied Biosystems, Waltham, MA, USA). The synthesized cDNA was used as a template for amplification with specific primers for *GAPDH* (endogenous control), *BAX*, *BCL-2*, *SOX2*, *SOX4*, *SOX9* and *SOX18* (Table 1). For quantitative PCR analysis, we used Power SYBR Green PCR Master Mix (Applied Biosystems, Waltham, MA, USA) in 7500 Real Time PCR Systems (Applied Biosystems, Waltham, MA, USA). All samples were measured in triplicate, and the mean value was considered. The relative expression level of analyzed genes was determined using comparative quantification algorithm where resulting ΔΔCt value was incorporated to determine the fold difference in expression (2^−ΔΔCt^).

### 4.8. Zebrafish Maintenance

Adult zebrafish (*Danio rerio*), wild type strain (Tübingen), were housed in a light and temperature controlled facility at 28 °C in 14 h:10 h light/dark cycle, and fed twice daily with commercially available dry flake food (TetraMin™ flakes; Tetra, Melle, Germany) and once a day with live brine shrimp (*Artemia nauplii*). Zebrafish embryos were collected from pairwise mating adults and kept and handled in egg water (containing 0.17 mM KCl, 0.33 mM CaCl_2_, 0.33 mM MgSO_4_, and 5 mM NaCl). All experiments involving zebrafish were performed in compliance with the European directive 86/609/EEC and the ethical guidelines of the Guide for Care and Use of Laboratory Animals of the Institute of Molecular Genetics and Genetic Engineering, University of Belgrade.

### 4.9. Anticancer Activity Evaluation in PANC-1-Zebrafish Xenografts

Prior to microinjection PANC-1 cells were stained with CellTracker™ RedCMTPX (Thermo Fisher Scientific, Waltham, MA, USA). The stain was dissolved in DMEM at final concentration of 2 μM. Cells were trypsinized, counted and resuspended in the stain solution, giving the concentration of 3 × 10^7^ cells/mL, and incubated for at least 1 h at 37 °C. Zebrafish embryos were manually dechorionated before microinjection on 48 h post fertilization (hpf). Number of injected cells was determined by applying the injecting volume on a microscope slide and counting (approximately 150 cells in 5 nL). Using a pneumatic picopump (PV820, World Precision Instruments, Sarasota, FL, USA) 150 cells were injected into the yolk sac in every anesthetized embryo. Embryos were then incubated for 1 h in clean embryo water at 28 °C to recover. Alive embryos were selected, transferred to a 24 well plate (10 embryos per well, 30 embryos in total per treatment) and treated with 0.5 μM or 1 μM of complex C1. DMSO was used as a negative control. Upon 120 hpf xenografts were photographed using Olympus BX51 fluorescent microscope with CytoVision Inksoftware (Leica Microsystems, Wetzlar, Germany). To determine the relative tumor size after complex C1 treatment, fluorescence intensity was measured using an image analyzing software Fiji (ImageJ 2.0.0) and compared to DMSO treatment.

### 4.10. In Vivo Hepatotoxicity Test

Complex C1 has been examined for the possible hepatotoxicity in transgenic *Tg*(*-2.8fabp10a*:EGFP) zebrafish embryos with the liver cells expressing green fluorescent protein (GFP). Complex C1 was tested at two different concentrations (0.5 and 1 µM). DMSO (0.25%) was used as a negative control. Zebrafish embryos have been treated from the 72 hpf stage (when the liver is fully functional, vascularized and has started the metabolic transformation of the absorbed compounds) to 120-hpf stage. After 48 h of treatment, the embryos were evaluated for the hepatotoxicity endpoints by fluorescent microscopy. The liver toxicity was assessed in relation to the control group according to the following monitored parameters: (i) liver fluorescence and size; (ii) liver necrosis; (iii) yolk resorption; (iv) liver area index (the ration between liver area and embryonic lateral area × 100%) as predictive parameters the hepatotoxicity described in literature [51]. The experiment was performed using 10 embryos per concentration. The liver area index was determined for five randomly selected embryos by ImageJ program. 

### 4.11. Statistical Analyses 

Statistical analyses were performed with the SPSS statistical software. The data represent means ± SEM from at least three independent experiments. Student’s *t* test was used to calculate *p* values (* *p* ≤ 0.05).

## Figures and Tables

**Table 1:** Primers used for qRT-PCR.

GAPDH F	5′-GCC TCA AGA TCA TCA GCA ATG C-3′
GAPDH R	5′-CCA CGA TAC CAA AGT TGT CAT GG-3′
SOX9 F	5’-CTT CTG AAC GAG AGC GAG A-3’
SOX9 R	5’-CTG CCC GTT CTT CAC CGA CTT C-3’
SOX4 F	5′-CCA AAT CTT TTG GGG ACT TTT-3′
SOX4 R	5’-CTG GCC CCT CAA CTC CTC-3′
qSOX2 F	5′-CCC CTG GCA TGG CTC TTG GC-3′
qSOX2 R	5′-TCG GCG CCG GGG AGA TAC AT-3′
SOX18 F	5′-TTC CAT GTC ACA GCC CCC TAG-3′
SOX18 R	5′-GAC ACG TGG GAA CTC CAG-3′
BAX F	5′-TGG CAG CTG ACA TGT TTT CTG AC-3′
BAX R	5′-TCA CCC AAC CAC CCT GGT CTT-3′
BCL-2 F	5′-TCG CCC TGT GGA TGA CTG A-3′
BCL-2 R	5′-CAG AGA CAG CCA GGA GAA ATC-3′

## Data Availability

The data presented in this study are available on request from the corresponding author.

## Sample Availability

Samples of the compounds used in this paper are available from the authors.
